# The Chairside Periodontal Diagnostic Toolkit: Past, Present, and Future

**DOI:** 10.3390/diagnostics11060932

**Published:** 2021-05-22

**Authors:** Tae-Jun Ko, Kevin M. Byrd, Shin Ae Kim

**Affiliations:** ADA Science & Research Institute, American Dental Association, Gaithersburg, MD 20879, USA; kot@ada.org (T.-J.K.); byrdk@ada.org (K.M.B.)

**Keywords:** periodontal diseases, oral diagnosis, dental equipment, periodontal probe, diagnostic imaging, biomarkers

## Abstract

Periodontal diseases comprise a group of globally prevalent, chronic oral inflammatory conditions caused by microbial dysbiosis and the host immune response. These diseases specifically affect the tooth-supporting tissues (i.e., the periodontium) but are also known to contribute to systemic inflammation. If left untreated, periodontal diseases can ultimately progress to tooth loss, lead to compromised oral function, and negatively impact the overall quality of life. Therefore, it is important for the clinician to accurately diagnose these diseases both early and accurately chairside. Currently, the staging and grading of periodontal diseases are based on recording medical and dental histories, thorough oral examination, and multiple clinical and radiographic analyses of the periodontium. There have been numerous attempts to improve, automate, and digitize the collection of this information with varied success. Recent studies focused on the subgingival microbiome and the host immune response suggest there is an untapped potential for non-invasive oral sampling to assist clinicians in the chairside diagnosis and, potentially, prognosis. Here, we review the available toolkit available for diagnosing periodontal diseases, discuss commercially available options, and highlight the need for collaborative research initiatives and state-of-the-art technology development across disciplines to overcome the challenges of rapid periodontal disease diagnosis.

## 1. Introduction

Periodontal diseases are a diverse group of chronic oral inflammatory conditions caused by microbial dysbiosis and the host immune response. In a recent study of global disease burden, periodontal diseases rank 11th of prevalent diseases in the world [1]. According to the new criteria presented in the 2017 World Workshop, periodontal diseases can be classified into dental biofilm-induced gingivitis, non-dental biofilm-induced gingival disease, necrotizing periodontal disease, periodontal manifestations of systemic disease, and periodontitis [2,3,4]. For periodontitis, the disease severity, extent, and progression are further classified based on multi-dimensional staging and grading systems [5]. Due to the heterogeneous clinical presentation of periodontal diseases, clinicians conduct multiple diagnostic analyses at the chairside—often tooth-by-tooth—to make an accurate diagnosis of both stage and grade. Several risk factors that may influence the condition, e.g., smoking, diabetes, obesity, stress, and genetic susceptibility, are also systematically examined for a more comprehensive diagnosis.

Clinical diagnostic analyses are generally based on the signs and symptoms of gingival inflammation and periodontal tissue destruction. The major diagnostic tools commonly used in the clinics are clinical observation (exam, photography), periodontal probing, and radiography. The tooth-supporting tissues, like the gums (i.e., gingiva), are observed for color changes, pain, edema, and positional changes, whereas the teeth themselves are examined manually for loosening and other direct signs of damage [6]. Moreover, the presence of plaque and calculus on teeth are examined. The use of the periodontal probe in the periodontal pockets around teeth provides information on pocket depth, periodontal tissue loss (clinical attachment loss), and presence of bleeding upon probing (i.e., a stimulating event). When these clinical observations and measurements with the probe are combined with radiographs, the pattern and extent of alveolar bone loss can be accurately evaluated. Only after collectively analyzing these data can the clinician ascribe the disease’s stage and grade, which aids in planning treatment [7].

Although the clinical diagnostic analyses inform both periodontal disease diagnosis and treatment, they are also inherently limited in that they can only assess disease history but not disease activity [8]. This is because once periodontal disease initiates, it does not follow a linear progression and can be characterized by periods of activity and remission [9,10]. To compensate for this limitation, many oral biomarkers from either oral fluid (saliva, gingival crevicular fluid (GCF)) or oral rinse have been developed for periodontal diagnosis, including host proteins, bacteria/bacterial products, ions, volatile compounds, and additional genotypic/phenotypic markers [11,12,13,14,15]. This remains a nascent field, but the incorporation of these biomarkers is expected to provide current disease activity and risk factors associated with the disease, allowing early and accurate diagnosis [16]. In this review article, (1) we summarize the current chairside periodontal diagnostic toolkit focusing first on the commercially available probing tools for the advanced clinical examination of the gingival sulcus, (2) we present advances in dental imaging technology (from two-dimensional to three-dimensional) for measurement of periodontal bone and tissues, (3) we briefly introduce the chairside biomarker technology currently on the market, and (4) we lastly summarize the future of chairside diagnosis and propose future research.

## 2. Diagnosis with the Available Chairside Probing Tools

### 2.1. Pocket Depth and Clinical Attachment Loss

**Figure 1:**
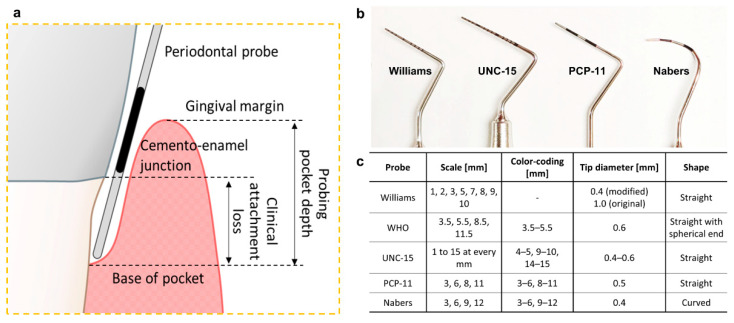
(**a**) Schematic presenting the measurement of PD between the tooth and gingiva using a periodontal probe with distance markings. (**b**) Various types of commonly used manual periodontal probes and (**c**) specifications [19].

Since chronic periodontal inflammation can lead to a loss of the supporting periodontium, measuring the loss of this attachment has been a key criterion for classification of the disease stage and grade and a strong predictor for future tissue destruction and thus disease progression [17]. Generally, a periodontal probe is used to measure both clinical attachment loss (CAL) and pocket depth (PD) (Figure 1a). CAL is defined as the distance from the base of the pocket (coronal end of junctional epithelium) to the cementoenamel junction (CEJ) of the tooth (hard tissue reference). In contrast, PD is the distance from the base of the pocket to the gingival margin (soft tissue reference). Both CAL and PD have been used as the defining periodontal disease analysis because each can be used to record changes in the periodontal condition over time. However, the PD quantifies the tissue loss without accounting for gingival margin level changes observed in gingival recession or overgrowth, and while more time-intensive to measure, CAL is a considered better diagnostic parameter to quantify the loss of periodontal attachment. Despite this, PD has been commonly used and is still recorded by general dentists and periodontists [6,18]. The 2017 World Workshop has now standardized that CAL between teeth (the interdental CAL) determines the periodontal disease classification [5].

There are many types of commercially available periodontal probes; however, manual probes remain the gold standard in dental clinics for measuring CAL and PD. The manual probes consist of a handgrip and probe tip. The tip design can range in length (12–16.5 mm in length), thin (0.4~0.6 mm diameter), and shape of the working end (spherical end, domed end, and a flat end with rounded corners) that can be inserted into the base of the pocket. In addition, various types and colors of graduated scales are displayed on the probe tip surface to help the manual reading of CAL and PD in millimeters (Figure 1b,c). For standardization of the probe, a general requirement for manual stainless-steel periodontal probes was specified by the International Organization for Standardization (ISO 21672-1 and -2:2012) [19,20]. However, even with these standardizations and specialized training, manual periodontal probe measurements can vary significantly due to inter-examiner differences in the probing force, probing angle, reading variations when reading manual scales, clinician proficiency, and the unpredictable anatomy of periodontal pockets, especially as the disease progresses [21].

Several attempts have been made to correct these inconsistencies. For example, force-controlled probes were designed that minimize the variation in probing force between clinicians have been developed. The force-controlled probes typically apply a force of 0.20 N to 0.25 N, allowing accurate diagnostic readings while minimizing patient discomfort [22,23]. These include True Pressure Sensitive probe (TPS probe, Ivoclar Vivadent, Schaan, Liechtenstein) and Pro-DenRx^®^ Sensor Probe (Den-Mat Holdings, Lompoc, CA, USA). These force-controlled probes are still operated manually but maintain constant probing force without electronic control. For example, the TPS probe has a sliding scale for visual guidance where two indicator lines meet at a specified force of 0.20 N. The visual-guided probing allows the clinicians to apply a constant force through the end of the probe. In addition to the visual guidance technique, electronic force-controlled probes were also introduced by Polson in 1980 [22]. Polson’s probe allowed an electronically controlled probing force that was constantly controlled at 0.25 N with an audio signal. This probe was further developed into the Yeaple probe, which is now used for dentinal hypersensitivity testing [24]. Despite the development of probing force control technology, measurement variation still exists due to the inter-examiner differences, and it is time-consuming to manually record and analyze a large amount of data accumulated in many sites (6 sites per tooth).

To reduce measurement variations caused by reading errors by eyes and save diagnostic time, automated digital readouts and computer-aided recording and analysis techniques have been applied. The Florida Probe^®^ (Florida Probe Corp., Gainesville, FL, USA) is one of the well-known computerized periodontal probing systems. The Florida Probe^®^ consists of a probe handpiece and sleeve, a displacement transducer, a footswitch, and a computer interface with automatic charting. Through the coil springs inside the handpiece, the probe provides a constant probing force of 0.15 N. Based on the constant probing force, the PD values are electronically measured with 0.2 mm precision and automatically transferred to the computer chart through the footswitch control or voice-activated software. Similar electronic systems that can measure the PD include the InterProbe™ (Dental Probe Inc., Ashland, VA, USA) and the pa-on Parometer^®^ (Orangedental GmbH & Co., Biberach, Germany). The InterProbe™ is designed to reduce probing pain with a flexible probe tip, and the pa-on Parometer^®^ is designed to be easier to use with graphical and acoustic feedback and an ergonomic wireless design. These advanced electronic probing systems eliminate readout and recording errors and save diagnostic time for PD measurements. However, despite the integration of computer-aided technologies and systemic automation, some clinical studies have not found significant differences in accuracy or measurement variability between the electronic and manual probes when measuring the PD or CAL [25,26,27,28]. Moreover, the additional training and costs limit the clinical use of these electronic probing systems.

Although several electronic periodontal probes have been successfully commercialized for automatic PD measurements, measuring CAL has been even more challenging to automate. The Foster-Miller probe (Foster-Miller, Inc., Waltham, MA, USA) was proposed to control the probing force and electronically detect the position of CEJ for CAL measurement. A ball tip of the probe is used to slide along the tooth’s surface at a controlled speed. Once the tip reaches CEJ, the speed of the tip rapidly changes then moves to the base of the periodontal pocket until it reaches the preset force. The CAL value can be electronically calculated and recorded based on the controlled speed, preset force, slide time, and acceleration time history. However, the prototype still requires further development, and it is not commercially available yet. 

Overall, incorporating new techniques (e.g., force-control, digitalization, and automation) has allowed the development of various types of periodontal probes, but only a few have been accepted in clinics due to their complexity in operation, low cost-effectiveness, and no significant improvements in accuracy nor reproducibility. The stainless-steel manual probes are still the most commonly used diagnostic tools for CAL and PD. To achieve better diagnostic accuracy and reproducibility, more innovation is still required. One such example can be developing multifunctional probes using nanotechnology and microfabrication in probe design and fabrication. Another innovation could be attempted in developing new probing protocols. Because the current probing protocol is done by probing six sites per tooth sequentially, it is time-consuming and uncomfortable for patients. Thus, more innovation and incorporation of new ideas will be the driving force for the future development of periodontal probes.

### 2.2. Bleeding on Probing

Gingivitis and periodontitis weaken the gums (i.e., pocket ulceration), so bleeding can occur during the probing of disease sites. For that reason, bleeding on probing (BOP) has been considered as a sign of periodontal disease and is evaluated as a numerical indicator called a BOP score [5,29]. The BOP score is assessed as a proportion of bleeding sites within six tested sites on all present teeth when stimulated by a standardized probe with a controlled force (0.2–0.25 N) to the bottom of the pocket. While the presence of BOP can be a poor predictor of periodontal disease activity, the absence of BOP is an excellent indicator of periodontal stability [30,31]. In the 2017 World Workshop, the BOP score was also recognized as a basic parameter that set thresholds for the diagnosis of gingivitis and assures the state of a healthy (BOP score < 10%) or gingivitis (localized: 10% ≤ BOP score ≤ 30%, generalized: BOP score > 30%) [32]. This was the first time that gingival health had been defined, serving as an important benchmark for future diagnostic and prognostic work.

To assess BOP, a binary readout (presence or absence) is performed, and manual periodontal probes are most commonly used. To test for BOP, clinicians use a manual periodontal probe to gently stimulate the tissue at the base of the periodontal pocket. Once bleeding occurs at the probing sites, the number of bleeding sites is quantified as a proportion of the total evaluated sites. When assessing gingivitis, dental floss or wooden interdental cleaners can also be utilized to assess BOP as an initial evaluation of a patient’s periodontal health [33,34]. However, some factors may influence BOP assessments, so they should be carefully considered [35]. For example, BOP can be less noticeable in smokers compared to non-smokers since smoking can cause gingival tissue keratinization and vasoconstriction [7]. Additionally, the manual probing force also affects BOP since the difference can lead to low reproducibility. In this case, force-controlled probes can be utilized for BOP assessment to possibly increase reproducibility [34]. So far, no electronic probe which automatically measures BOP score has yet to be commercialized. It is recently reported that hemoglobin (Hb) presence in GCF suggests slight tissue damage, even in healthy sites defined as an absence of BOP [36]. Therefore, the emergence of new probes capable of detecting the bleeding (or Hb) within the periodontal pocket in a quantitative way may help improve the utility of the analysis and increase the diagnostic sensitivity of periodontal diseases.

### 2.3. Tooth Mobility

Tooth mobility is an important physical feature of periodontal diseases. When periodontal diseases occur, it can cause bone resorption and damage to the supporting soft tissues. As a result, the structure that holds the teeth firmly in place is lost, and the teeth become mobile [37]. Tooth mobility is determined clinically by putting directional pressure on the tooth and observing its movement. The Miller index is the most commonly used manual method in which the tooth is held firmly between two instruments and moved back and forth [38]. For a more accurate and reproducible evaluation of the degree of tooth mobility, numerous techniques have been studied and tested. These include devices of electronic registration [39], microperiodontometer [40,41], dental holographic interferometry [42,43], laser vibrometer [44], piezoelectric transducer [45], resonance frequency analysis (RFA) [46], and non-contact vibration device [47,48]. 

Among various devices, the Periotest^®^M (Medizintechnik Gulden, Modautal, Germany) is an electronic wireless device for assessing periodontal disease parameters (including mobility) of teeth and osseointegration of dental implant [49,50]. The basic principle of the device is based on measuring the response reaction from a reproducible impact, which is applied to the center of the tooth surface. It measures contact duration per impact between the rod and the tooth while an electrically controlled rod percusses the tooth and then recoils. If there is any periodontal structural deviation in bone and/or soft tissue that is caused by any periodontal disease, it will be reflected in the contact duration. Another similar device, Implomates (Bio Tech One Inc., Taipei, Taiwan), percusses the tooth with a metal rod driven by an electromagnetic field and records the induced vibrations using a microphone with 50 Hz resolution. However, the output of these percussion tests lacks reproducibility since they undergo significant changes as recording position and angulation of the test device vary [51]. As a non-contact method, the Osstell IDx (Osstell AB, Gothenburg, Sweden) measures the implant stability (osseointegration) with non-invasive and non-destructive techniques. This product utilizes RFA with resonance frequency ranging from 3000 to 8500 Hz for an implant stability quotient of 0–100. The device consists of a transducer, a computerized analysis module, and an excitation source [52,53]. Although the Osstell IDx is optimized for osseointegration instead of natural tooth mobility test, the device enhances patient comfort and increases reproducibility [51]. Therefore, it is expected that the tools of tooth mobility test are also developed based on a non-contact vibration technique to minimize the variations depending on the test position and operating condition of the device.

### 2.4. Plaque and Calculus

Over 700 species of oral microbiome inhabit the human oral cavity, and about 400 species are found in the subgingival plaque [54]. Periodontal disease is driven by dysbiotic, subgingival biofilms in susceptible hosts. These subgingival species secrete various compounds that can cause tooth decay and periodontal tissue inflammation [55,56]. Moreover, this irritation causes an inflammatory reaction that can lead to biofilm-induced gingivitis and periodontal disease [4,32]. When the plaque is not removed effectively, calcium phosphate mineral salts in the saliva combine with the plaque between and within remnants of formerly viable micro-organisms to develop calculus [57]. Calculus (colloquially referred to as “tartar”) is also well known as a biofilm-retentive factor. Thus, teeth with calculus show a significantly higher risk of attachment loss than teeth without calculus [58].

To examine the presence of plaque and calculus, sharp explorers or manual periodontal probes have been typically used. Like with BOP, clinicians binarily assess the presence or absence of this accumulated material. There are indices used in research to grade plaque on more nuanced scales; however, these are not widely implemented in the clinic. However, the traditional tactile assessment of the subgingival root surface without visual accessibility lacks accuracy, specificity, and reproducibility. Since the average percentage of accurate detections of clinically identifiable calculus depends on the clinical expertise, the subgingival debridement may lead to varying degrees of residual calculus, removal of root cementum, or both [59,60].

To overcome these shortcomings in identifying the calculus, several different technologies have been incorporated into the probe platform. The DetecTar (NEKS Technologies Inc., Quebec, Canada) identifies subgingival calculus by evaluating the characteristic optical signals on root surfaces and detecting spectro-optical differences between calculus and the tooth surface. When the subgingival calculus is irradiated with a specific wavelength light, it results in the production of a characteristic spectral signature caused by absorption, reflection, and diffraction. These spectral signals are sensed by an optical fiber and converted into an electrical signal for computer analysis. The shape and dimension of the DetecTar probe tip are similar (0.45 mm diameter) to those of conventional periodontal probes. The system is also available as a portable cordless handpiece with a curved periodontal probe with millimeter scales to measure CAL and PD. As another tool, the Perioscopy (Zest Dental Solutions, Carlsbad, CA, USA), which is a miniature periodontal endoscope, is inserted into the periodontal pocket for subgingival visualization of the root surface with tens of magnifications (maximum 48× magnification). This endoscope-based system optically helps to identify the residual calculus spots during the examination. The Diagnodent (KaVo Kerr, Brea, CA, USA) is a pen-like probe that sends a safe, painless laser beam into the tooth to detect the autofluorescent signal from calculus lesions. The Diagnodent can measure a wide range of fluorescence intensities that are displayed on a digital display as a series of relative calculus detection values.

Furthermore, some products have combined the diagnosis and treatment functions for clinical convenience. The PerioScan (Dentsply Sirona, York, PA, USA) provides a diagnosis mode to detect calculus deposits and a treatment mode for the conventional ultrasonic debridement with different power levels. When the ultrasonic tip detects calculus on the tooth surface, blue light and an acoustic signal simultaneously are displayed on both the handpiece and the display to facilitate diagnosis. The Key Laser 3+ (KaVo Kerr, Brea, CA, USA) is another product that can conduct calculus detection and removal in a feedback-controlled manner. The automated device contains a 655-nm InGaAs diode laser for calculus detection and a 2940-nm solid-state erbium-doped yttrium aluminum garnet (Er:YAG) laser for calculus removal. The Er:YAG laser is activated or inactivated depending on the detected calculus level based on the feedback-controlled system. According to some literature, there are no statistically significant differences between the feedback-controlled laser debridement and the ultrasonic treatment [61,62]. The advantage of both systems is that the diagnostic and treatment modes can be used continuously on the same tooth surface without changing tools, but the specificity of the calculus detection still needs to be improved. Especially, false detection, in which irregularities on the root surface are erroneously recognized as calculus, remains a challenge to be solved.

### 2.5. Other Parameters with Diagnostic Potential

In addition to traditional clinical examinations, sensor-integrated probes also have been used to measure additional disease-related parameters. Here, we introduce two examples of chairside sensor-integrated probes based on auxiliary parameters: temperature and sulfide concentration. 

In general, the gingival temperature is increased by elevated blood flow and cellular/metabolic reaction as a host-response to inflammation. The vascular changes are accompanied during the inflammatory process (i.e., vasodilatation, increased vascular permeability, and increased blood flow), and the increased fluid transport in the inflamed site raises the temperature. Therefore, temperature evaluation in the specific gingival area can be clinically supported as an inflammation indicator [63,64,65,66,67]. Compared to a healthy site, a temperature rise of 0.7–3.0 °C was reported in the gingival sulcus (or gingival surface) with periodontal disease [63,64,65,66,67,68,69]. The PerioTemp^®^ probe (Abiodent Inc., Danvers, MA, USA) has been used in many clinical studies for subgingival temperature measurement [67,68,70]. Key benefits of using this probe include a rapid response time (<1sec), high accuracy (±0.1 °C), and high reproducibility. Its physical shape and dimensions, similar to conventional periodontal probes, allow the measurement of CAL, PD, and BOP as well as temperature. It also has a computerized thermometer that displays actual subgingival temperature (optional) and a risk level with two-color light indicators [21,67]. It has been reported that this probe can help in the early diagnosis of periodontal disease and detect disease activity by detecting subgingival temperature related to inflammatory changes [66,67]. In addition, the performance of the PerioTemp^®^ was reported to be comparable to that of an infrared thermometer (Thermoscan^®^ IRT 3520, Braun, Kronberg, Germany) with only about 0.18 °C in the mean difference for the measured gingival temperature [71]. Although many clinical trials evaluate periodontal disease progress or activity, the subgingival temperature change assessment has not been perceived as a periodontal disease evaluation method [68]. Determining the normal temperature distribution can be very complicated as there is a large variation between the patient, the location of the examination site, and the environmental conditions (e.g., respiratory airflow and ambient temperature). For the subgingival temperature to be recognized as a diagnostic parameter, further investigation is needed, including the development of a new material showing better reliability and reproducibility, the design of a new temperature probe that comprehensively considers various factors, the standardization of a reliable measurement procedure, and the accumulation of data showing clinical relevance.

It is well known that gram-negative bacteria are plaque-induced bacteria that generate sulfur-related substances as by-products from their metabolism. These substances are known to be volatile sulfur compounds (VSC), including hydrogen sulfide (H_2_S), methyl mercaptan (CH_3_SH), and dimethyl sulfide (CH_3_SCH_3_) [72]. When gram-negative bacteria invade the underlying connective tissue of the periodontium, an inflammatory reaction can begin and result in a tissue disruption [72]. Several clinical studies have reported that sulfide by-products are associated with periodontal diseases, especially including plaque-induced gingivitis [72,73,74,75,76,77]. Many sulfide detection tools have also been introduced, e.g., Halimeter^®^ (Interscan Corp., Simi Valley, CA, USA), Oral Chroma™ (Nissha FIS, Osaka, Japan), and Breathtron^®^ (Yoshida, Tokyo, Japan), but they are designed for halitosis sensing and unavailable for diagnosing of PDs in the gingiva sulcus and periodontal pockets. The Diamond Probe^®^/Perio 2000^®^ System (Diamond General Development Corp, Ann Arbor, MI, USA) is designed to detect sulfide levels in the gingival sulcus and periodontal pockets in real-time for gram-negative bacteria monitoring. In this system, a microscale sulfide sensor was incorporated into a modified Michigan O-type periodontal probe to measure CAL, PD, BOP, and sulfide levels. When the sensor-integrated probe tip encounters sulfides in the GCF, the system provides information in three ways: a four-color light bar, an audible tone, and a sulfide level [75]. According to multiple clinical studies, this sulfide probe demonstrated that the intra-pocket sulfide level is positively correlated with the progression and severity of periodontal disease and was higher in untreated subjects than maintenance subjects [72,74,78,79]. However, the sulfide probing system is not commonly implemented in the clinic. The key reason is that the newly introduced probe is still in the conceptual stage, and their cost-effectiveness does not offer a significant advantage for periodontal disease diagnosis. 

Since periodontal diseases are associated with systemic diseases, fast and accurate diagnosis is becoming more important. Periodontal probe systems have made various efforts to determine the PD and CAL with high accuracy and reproducibility. Additionally, to provide additional evidence in periodontal disease diagnosis, various sensors (e.g., mobility sensor, calculus sensor, temperature sensor, sulfide sensor) have been integrated into the probing tools. However, the probing tools commercialized so far are still at a very early stage of development. Therefore, ongoing developments of probing tools, innovative approaches, and collecting verifiable evidence will help in the early and accurate detection of the risk of periodontal diseases, disease onset, and monitoring of oral health conditions and systemic diseases (e.g., cardiovascular disease, diabetes, and inflammatory bowel diseases) [80].

## 3. Diagnosis with the Imaging Tools

### 3.1. 2-Dimensional Imaging with Radiography

Conventional two-dimensional oral radiography is the most widely used imaging method for diagnosing periodontal disease. The radiography minimizes patient discomfort and pain in a non-invasive manner [81,82]. Conventional radiography provides a two-dimensional (2D) image from a three-dimensional (3D) structure in the oral cavity and reflects the anatomy of the hard tissues, including bone, cementum, dentin, enamel, and calculus. The primary purpose of radiograph for periodontal disease assessment is to measure the level of alveolar bone together with the observation of the factors including bone level, bone destruction pattern, marginal contour, and extent of bone loss. 

**Figure 2:**
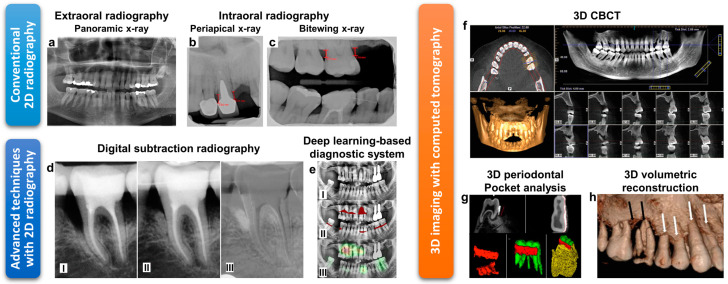
(**a**–**c**) Conventional 2D radiography. (**a**) Panoramic X-ray for capturing the entire mouth with an overall view. Reprinted with permission from [84]. (**b**) Periapical X-ray and (**c**) bitewing X-ray for evaluation of periodontal disease. Reprinted with permission from [85]. (**d**) Digital subtraction radiography for bone-regeneration assessment; (I) baseline radiograph, (II) after 12-month radiograph, and (III) subtraction of (II) from (I). Reprinted with permission from [86]. (**e**) Deep learning-based periodontal bone-loss diagnosis; (I) panoramic radiograph, (II) bone-loss lesion annotated by clinicians, and (III) bone-loss class activation map highlighted by the deep-learning-based system. Reprinted with permission from [87]. (**f**) CBCT software interface including pan-map (top-right), horizontal section (top-left), vertical sections (bottom-right) and 3D reconstructed model (bottom-left). Reprinted with permission from [88]. (**g**) The depth and volumetric measurement of the periodontal pockets using CBCT. Reprinted with permission from [89]. (**h**) 3D volumetric reconstructive CBCT image obtained from a patient with aggressive periodontitis. Reprinted with permission from [90].

The common types of dental 2D radiography are panoramic and intraoral X-ray. The panoramic X-ray captures the entire mouth with an overall view (a half-circle from ear to ear) in a single image (Figure 2a). Since it visualizes the entire area in a single film, including maxilla, mandible, and temporomandibular joints, it is useful for treatment planning of extractions, implants, and dentures. Also, it is a reliable tool in observation of alveolar bone loss caused by periodontal disease; however, this method is not suitable to diagnose the activity of periodontal disease since it hardly provides information about periodontal soft tissues [83].

Intraoral X-rays take an image with a radiographic film or a detector placed inside the mouth and are used in periapical views to provide precise and detailed information about each tooth and partial bone with various views. (Figure 2b). On the other hand, bitewing radiographs differ from periapical radiographs in that they are usually limited to capturing the image of the 3–4 upper and lower teeth in one area of the mouth. A horizontal bitewing radiograph is primarily used to detect bone height measurements along the tooth root, while vertical bitewings are used to evaluate bone loss (Figure 2c) [91]. The change of intraoral radiograph is used as important evidence of periodontitis progression. In addition, observation of furcation involvement based on intraoral radiographs can be utilized to evaluate the amount of bone loss and tissue destruction [82].

Since digital radiography was first introduced to dentistry in 1987, it has rapidly expanded to clinics by overcoming the shortcomings of traditional film-based radiography, such as time and space constraints for printing [92,93]. Moreover, radiography obtained with a digital detector can reduce the amount of radiation dose to the patient up to 90% compared with film-based radiography due to the high sensitivity of the digital imaging detectors [94]. Digital radiographs can be instantly displayed, stored, printed, and sent to other electronics by use of a digital image capture device and a computer. Based on the digital radiographs, the changes in bone density or volume can be easily recognized by the contrast difference, i.e., lighter area refers to large bone density, and darker area refers to bone loss. Furthermore, computer-aided image processing software enables high precision analysis allowing easy assessment of disease severity and progression. Digital subtraction radiography (DSR) is one of the representative technologies that use digitized radiographs. DSR can record and superimpose two images of the same object obtained at different time points, allowing for a visualized direct comparison (Figure 2d). An algorithm can then subtract the image intensities from the identical pixel and automatically highlight the area that has any differential. This technique allows the clinicians to easily diagnose tissue or bone loss in a specific area by fading out of unchanged areas. One form of subtraction radiography widely utilized in the research and clinic is computer-assisted densitometric image analysis (CADIA). It uses a computerized video camera and an image analyzing processor to measure the light that is transmitted through the radiographs. The light signals that are converted to greyscale images can be mathematically processed. The quantitative information results in two radiographs of the same anatomical location compared via superimposition [95]. This then allows comparison and highlights any area with changes in density possibly caused by bone density change or the existence of furcation involvement. The CADIA system also evaluates bone regeneration in the extraction socket or the bone density changes in a furcation [96,97]. Furthermore, in a recent study, deep learning technology using artificial intelligence has been applied to automatically detect periodontal bone loss in a 2D dental radiography (Figure 2e) [98,99].

Even though 2D imaging has been widely accepted as a standard imaging technique for an oral health assessment, it contains some inevitable limitations while the 3D structural information is presented in a 2D plane image by superimposition. Generally, image distortion and blurring occur by the superimposition, hindering the accurate assessment of delicate bone structures. For instance, the 2D imaging-based diagnosis of bone destruction caused by periodontal disease tends to underestimate the actual severity [100]. To overcome these limitations, various 3D-based imaging techniques that can reveal complex bone structures have been introduced. These efforts help to determine a more accurate diagnosis and plan for periodontal disease treatment. 

### 3.2. 3-Dimensional Imaging with Computed Tomography

Computed tomography (CT), invented in 1972, has been used in oral health assessment as an extraoral radiography method [82]. CT can build 3D image models without superimposition of structures by assembling 2D cross-sectional images obtained from all planes of interest. A study demonstrated that the alveolar bone height and intrabony pockets could be precisely evaluated using CT assessment [101]. Although CT offers high-quality 3D images with improved accuracy, increased radiation exposure and high cost are concerns in adopting this periodontal disease diagnosis technique in dental clinics [102].

As an alternative option to conventional CT technology, a cone-beam computed tomography (CBCT) has been introduced. The dental CBCT system allows a conical X-ray beam to capture data from rotating around the patient with a ten-fold reduction in radiation than conventional CT [103]. These data are then used to reconstruct 3D images of the patient’s dental and maxillofacial areas (transverse section, sagittal section, and buccal section) using software (Figure 2f). These reconstructed 3D models help the clinician clearly understand the relationship between the lesion’s size and the surrounding anatomical structures. It is also used to evaluate the progress of the disease and establish a treatment plan. The 3D dental CBCT technology is more advantageous in bone and periodontal pocket assessment than the conventional 2D radiography (Figure 2g,h) [89,90,104]. The advantages and specifications of CBCT compared to the conventional 2D radiography are summarized in Table 1. Up to date, various CBCT equipment have been commercially available, including CS 9300 (Carestream Health, Rochester, NY, USA), Orthophos SL 3D (Dentsply Sirona, York, PA, USA), i-CAT FLX (KaVo Kerr, Brea, CA, USA), ProMax^®^ 3D (Planmeca, Helsinki, Finland), PaX-i3D (Vatech, Hwaseong, South Korea), Prexion3D (Prexion, San Jose, CA, USA).

For a series of axial cross-sectional images, tuned aperture computed tomography (TACT) has also been utilized. It is designed to produce holographic images with 3D views of teeth and pathology [113]. This equipment effectively detects the location of periodontal bone gain or loss, alveolar contour, and even recurrent caries [113,114]. Cone-beam volumetric tomography (CBVT) is another CT-based technique that offers high resolution and accuracy for assessment after regenerative therapy (e.g., bone fill or defect resolution) [115]. Quantitative computed tomography (QCT) offers more detailed bone mineral density information with precise 3D anatomic localization of bone density assessment [116]. Moreover, micro-focus CT can provide adequate information about the alveolar bone structure and tooth morphology based on its fine spatial resolution (<10 µm). This technique enables faster 3D image reconstruction and allows minimal side effects. In addition, 3D imaging of interface in bone-implant has been reported using micro-focus CT [117].

Although radiation doses from advanced CT are generally lower than conventional CT, 3D imaging with CT typically delivers more radiation than 2D radiography. Therefore, the American Dental Association (ADA) and the US Food and Drug Administration (FDA) recommend that clinicians perform dental radiography, including dental CBCT, only when necessary to diagnose or treat the disease [104]. Efforts to reduce radiation exposure and increase image resolution will continue in the future. Table 1 summarizes the characteristics of 2D radiography and 3D CBCT, including effective radiation doses for radiographic examination.

### 3.3. Ultrasonography

Diagnostic imaging with non-ionizing radiation can non-invasively evaluate the structure and function of the human body without the risks associated with ionizing radiation. Ultrasonography is a diagnostic imaging technique that exhibits the internal tissue structure using reflections or echoes of ultrasound signals and is thus, non-ionizing. The ultrasonic image uses a small probe to send ultrasound pulses (1~20 MHz in medical diagnosis) to the tissue and displays the acoustic impedance of a 2D cross-section of tissue based on the reflective properties of each tissue. 

In dentistry, ultrasound was first used for diagnosis in 1963 [118]. Baum et al. tried to observe the tooth’s internal structure using a 15 MHz transducer, but the image clarity and quality were too poor to confirm a detailed structure. Afterward, based on advanced imaging technology and software development, ultrasound devices for intraoral diagnosis were developed, including Krupp SDM^®^ (Krupp Medizintechnik, Essen, Germany), SonoTouch (Ultrasonix Medical Corporation, Richmond, Canada), IO3-12 (Alpinion, Seoul, South Korea), and UltraSonographic Probe (US Probe, Visual Programs Inc., Ashland, VA, USA). These instruments have been clinically applied to measure gingival lesions, tooth fractures, soft tissue lesions, maxillofacial fractures, alveolar bone defects, and gingival thickness [82,88,119,120,121,122]. In particular, the US Probe adopts the periodontal probe platform with the tapered tip, which produces a narrow ultrasonic beam profile (~0.5 mm) using a 1–20 MHz transducer. These ultrasonic waves are carried through an area created by a small water stream into the periodontal pocket. The US probe can provide PD information without probing pain as well as gingival tissue images with sufficient signal strength and penetration depth along the gingival line [123].

So far, the use of ultrasound has not been widely adopted for disease diagnosis in dentistry as an alternative method of radiography. However, recent studies demonstrated that high-resolution ultrasonography clearly shows the cross-sectional morphological images of the periodontal tissues, suggesting the possibility of applying ultrasound for periodontal imaging. For example, Nguyen et al. reported that the alveolar crest level, the CEJ location, and the alveolar crest’s thickness measured from ultrasonography presented less than 10% of difference compared with those obtained from CBCT, proving ultrasound can provide clinically reliable data [124]. Although ultrasound application in dentistry is still in its infancy, it is believed to have massive potential as a diagnostic tool due to its various benefits, e.g., portability, cost-effectiveness, free-of-ionizing radiation, and real-time observation.

### 3.4. Magnetic Resonance Imaging

Based on its excellence for soft tissue imaging, magnetic resonance imaging (MRI) has been considered a promising diagnostic tool in dentistry since 1981 [125]. MRI has exhibited outstanding ability in soft tissue imaging among non-invasive diagnostic techniques, allowing the MRI to have expanded its application for disease diagnosis in various medical fields. MRI forms an image by measuring a pattern in which a hydrogen atom nucleus absorbs and emits an electromagnetic wave in a magnetic field in the 0.2 to 7 Tesla (T) range [126]. Since hydrogen is found in abundance as a form of water in soft tissue, MRI can provide high contrast sensitivity to soft tissue, in contrast with CBCT that is specialized in hard tissue imaging. A study conducted by Assaf et al. reported the visibility of osseous and tooth-related structures using MRI. The result demonstrated that the region with soft tissue (e.g., pulp chamber, apical foramen, mandibular nerve canal) presented good visibility, while the enamel-dentin junction, cementum-dentin junction, and periodontal space were challenging to visualize [127].

In recent years, MRI has been utilized for temporomandibular joint or jaw lesion observation, pulp vitality evaluation, as well as endodontic treatment and implant planning [128,129]. In contrast, 2D radiography or CBCT specialized in the depiction of hard tissue structures such as alveolar or mandible bone, MRI is adequate in visualization or differentiation of the soft tissues. Therefore, it is capable of detecting the histopathological change that occurs in the gingiva during the early stage of periodontal disease [130,131,132]. A recent comparison study between MRI and CBCT demonstrated that MRI is superior to CBCT in the visualization of periodontal space and cortical/trabecular bone. Primarily, MRI provided significantly better images for periodontal structures like lamina dura as well as bone structure (e.g., cortical and trabecular bone), suggesting the high potential capability of MRI in periodontal disease detection and periapical lesion observation [132]. Geibel et al. also reported that MRI could be more advantageous for accurate observation of apical periodontal lesions compared to CBCT imaging in terms of characterization and identification of periapical cysts and granuloma [133,134]. MRI also has been employed for furcation involvement observation. The furcation involvement is one of the symptoms of periodontal disease and occurs with bone resorption into the furcation area of a tooth root. It has been typically evaluated using a curved periodontal probe in clinical assessment, but it is challenging to accurately evaluate the severity due to complex root morphology and limited accessibility of the probe [135]. In a study conducted by Alexander et al., MRI was proven to demonstrate higher accuracy and reliability in imaging of furcation involvement in maxillary molars compared to CBCT, allowing the accurate classification of furcation involvement [135]. Based on several advantages in soft tissue imaging, MRI remains a promising imaging tool for the diagnosis and treatment planning for periodontal disease.

Even though MRI devices were successfully applied for soft tissue imaging, the imaging of hard tissues such as teeth, dentin, and enamel, which have low water content, is inevitably restricted [136]. This poor visibility for hard tissue has been regarded as one of the obstacles to the clinical implementation of MRI. However, a recent study suggested overcoming the limitation of hard tissue imaging using MRI and demonstrating that using MRI can be effective for both soft tissue and hard tissue observation. Algarin et al. designed a special-purpose MRI scanner (DentMRI-Gen I) capable of producing high-quality combined images of soft and hard biological tissues at sub-Tesla fields (260 mT) [137]. However, simultaneous imaging of soft and hard tissues using MRI requires more clinical validation, so there are remains opportunities for technology development. Some challenges that remain include the accessibility of equipment due to its high cost as well as the discomfort of its use during long scanning time. The magnetic field may also cause metal-based implants (e.g., hearing aid, cardiac pacemaker, or electrical stimulator) to malfunction and possibly result in an injury. Lastly, higher technological expertise is required for MRI utilization than for other imaging tools, which should be addressed to expand MRI application.

### 3.5. Digital Dental Photography

Digital dental photography is a type of macro-photography that provides information on the surface of the oral cavity as part of the patient workup. Recently, there have been reports of using digital photographs for examination, diagnosis, and treatment planning of oral diseases [138]. With the advent of digital single-lens reflex (DSLR) cameras, digital photography has become photographic documentation to accurately record clinical manifestations of the oral cavity. Correct color rendition with accurate exposure control and sufficient resolution is essential to record salient features of both soft and hard tissue details [139]. In specific, the correct color rendition of the photographs is an excellent method for distinguishing between healthy and diseased soft tissue, including white patches, inflammation, ulceration, carcinoma. In addition, digital photographs of sufficient resolution can distinguish between healthy and diseased tissue by providing morphological information such as gingival clefts and recession. Although digital photographs have not yet been widely used for diagnosing periodontal diseases, their use in virtual patient care is foreseen [140]. In addition, advances in the digital camera, optimization of image processing, and automation of machine learning are expected to further expand the use of digital dental photography in the future.

### 3.6. Intraoral Scanners

Intraoral scanners were developed to replace conventional dental impressions used in prosthodontics, orthodontics, and restorative dentistry [141]. With the advancement in computer-aided design and digital photography technologies, the digital intraoral scanner has been successfully expanded the application based on its several advantages. It projects a light source onto the object to be scanned and sends morphological information to a connected computer system to render the 3D model with a digitized form. While conventional dental impressions require complicated steps, the intraoral scanner can reduce the time to obtain the information, which relieves patients’ pain and discomfort as well as clinicians’ burden. Also, it is capable of real-time scanning and visualization, allowing rapid diagnosis and communication based on the digitized result without concern of potential deformation [142].

A few recent studies suggested that the digital assessment method using the intraoral scanner is possible to apply in gingiva health assessment [143]. For example, oral scanners were used to measure periodontal defects and evaluate tooth mobility, one of the parameters evaluating periodontal disease [144,145]. Zhang et al. also reported that gingival volume change in patients with periodontitis after therapy could be recorded using an intraoral scanner. The results were positively correlated with other parameters, including PD, bleeding index, and keratinized gingival width [143]. Intraoral scanning can also assess gingival health or tissue regeneration by identifying the color difference in soft tissue. Considering that there is a color difference between keratinized and nonkeratinized tissues, it is possible to evaluate the keratinized mucosa’s dimensions [146]. In a study by Lee et al., they used a 3D intraoral scanner with LED as a light source that is capable of color recognition and claimed that the digital scanning could more accurately measure the keratinized tissue width than using a periodontal probe [147]. This digital oral scanning technology enabling color recognition is expected to expand its application as a simple and promising tool for gingival health assessment and sulcus detection in the future [148]. To date, several intraoral scanning devices have been commercially available, such as TRIOS 4 (3Shape, Copenhagen, Denmark), iTero Element^®^ (Align Technology, San Jose, CA, USA), CEREC Omnicam (Dentsply Sirona, York, PA, USA), and Emerald™ (Planmeca, Helsinki, Finland).

### 3.7. Endoscopic Capillaroscopy

Another imaging technique for periodontal health assessment is endoscopic capillaroscopy. This technology can image and record the microvasculature of the periodontal pocket and gingival crevice in vivo by inserting a sub-millimeter-sized optical fiber into the periodontal pocket. It uses a green light with a wavelength of 520 nm for illumination absorbed by both oxygenated and deoxygenated blood. The blood vessels with red blood cells appear dark on a green background, allowing the high-resolution imaging of the periodontal pocket microcirculation [149]. Although no cases have been reported for actual periodontal disease evaluation, it is expected that the combination of capillary examination and optical fiber technology can be used to observe the size change of microvessels caused by periodontal disease.

## 4. Chairside Biomarker Detection

The heterogeneity of periodontal disease onset, patterning, activity, and treatment response has hindered the accurate and reliable diagnosis of periodontal tissue infection. For this reason, the symptom-based diagnosis techniques, e.g., use of periodontal probes or dental imaging, can only provide the status about previous damages caused by periodontal disease, but they are limited to obtain detailed information about disease activity that is required for personalized treatment planning. However, with a recent understanding of pathophysiology in periodontal diseases, various biomarkers (e.g., biochemical-, microbial-, or genetic-based) have been suggested as having potential for improving diagnosis or outcomes for better understanding of disease activity [8,11,12,13,32]. As inflammation further processes due to periodontal disease, several types of mediators are released into GCF and saliva as well as other oral rinses, thus they can be utilized as indicators through biomarker assays. These biomarker assays are believed to help identify periodontal pathogens as a readout of disease activity and for assessing periodontal disease severity. In addition, these methods are expected to allow the clinicians to evaluate the prognosis after treatment for any future treatments [11,12,150,151]. Although an ideal biomarker for periodontal disease has yet to be identified, biomarkers that measure disease pathogenesis or bio-components linked to tissue destruction, host defense mechanisms, and bacterial metabolism during disease progression are actively being studied [8,152].

### 4.1. Biochemical Assay Kits

Efforts to develop a chairside biomarker assay kit for rapid periodontal disease diagnosis are continuing worldwide. For example, biochemical assay kits like PerioSafe^®^ PRO DRS (Dentognostics GmbH, Solingen, Germany) and ImplantSafe DR^®^ (Dentognostics GmbH, Solingen, Germany), which can measure active matrix metalloproteinase-8 (aMMP-8) in dental and medical clinics, were recently commercialized in Europe. MMP-8 is a major mediator of tissue destruction in periodontitis, and a correlation between increased MMP-8 activity and progressive loss of connective tissue attachment and osteolysis has been demonstrated through numerous clinical studies [11,12,153,154,155]. The PerioSafe and ImplantSafe tests qualitatively measure aMMP-8 levels in oral rinse and GCF, respectively [156]. In addition to the qualitative analysis, the PerioSafe and ImplantSafe test sticks can quantify the amount of aMMP-8 in ng/mL units within 5 min. using the automated digital device, ORALyzer^®^ (Dentognostics GmbH, Solingen, Germany). According to recent reports, the device with aMMP-8 assay kits is useful for distinguishing between active and inactive sites with fast and easy analysis and detecting asymptomatic, ongoing periodontitis before clinical and radiographic signs appear [153,157]. 

Other chairside biomarker assay tools using aspartate aminotransferase (AST) were also developed. AST is an enzyme that is abundant in human gingival epithelial cells and gingival fibroblasts. Upon cell death, a large amount of AST is released from the cell cytoplasm into the GCF thereby, the level of AST in GCF can be used as a strong indicator of gingiva tissue destruction [158]. PerioGard (Xytronyx, Inc., San Diego, CA, USA) and PocketWatch (Steri-Oss Inc., Yorba Linda, CA, USA) are the chairside diagnostic tools that diagnose periodontal disease by the assessment of AST level in GCF. In both products, the activity of AST is evaluated based on the enzymatic catalysis reaction by comparing the color of the collected GCF from patients with that of the controlled AST positive group.

In addition, to assess the risk of oral disease, a chairside saliva testing device using test strips with multiple (6 to 7) indicators has been introduced in Japan. The SillHa (ARKRAY Inc., Kyoto, Japan) and the Salivary Multi Test^®^ (SMT^®^; Lion Corp., Tokyo, Japan) can measure multiple saliva indicators (blood, leukocytes, and proteins) related to gingival health [159]. Both products consist of test strip kits and an automated wavelength reflectometry device that reads color changes on the test strips within a few minutes (~5 min). These toolkits of chairside biomarker assay in the market are summarized in Table 2.

### 4.2. Microbiological Assay Kits

Microbiological diagnostic tools also have been widely studied. It has been found that over 700 species of oral microbiome related to oral disease, and some are significantly associated with periodontal disease and can be used as microbiological biomarkers for the disease diagnosis [54]. To date, the onset of periodontitis is related to pathogens including *Porphyromonas gingivalis* (Pg), *Treponema denticola* (Td), *Tannerella forsythia* (Tf), *Actinobacillus actinomycetemcomitans* (Aa), *Prevotella intermedia* (Pi), *Fusobacterium nucleatum* (Fn), and *Filifactor alocis* (Fa) [158]. These bacteria generally co-locate in periodontal pockets with a wide distribution and increased numbers suggesting that these can be potential biomarkers. Some periodontal pathogens, including Pg, Td, Tf, and some *Capnocytophaga specie,* produce bacterial trypsin-like proteases by utilizing the hydrolysis reaction of BANA (N-Benzoyl D-L Arginine -2 Naphalamide) in the biofilm. Thus, based on this hydrolysis reaction, the BANA-Enzymatic test™ kit (Ora Tec Corporation, Manassas, USA) is developed as rapid and reliable chairside diagnostic tests. The Evalusite (Eastman Kodak company, Rochester, NY, USA) is a rapid microbiological assay kit that detects three recognized pathogens: Aa, Pg, and Pi. By collecting a subgingival plaque, the kit detects the presence of the pathogens based on an antibody-bounded sandwich-type enzyme-linked immunosorbent within 10 min. As a chairside diagnostic platform, OMNIgene (DNAgenotek^TM^, Ottawa, ON, Canada), iai PadoTest (IAI AG, Zuchwil, Switzerland), MyPerioPath^®^ (OralDNA Labs, Eden Prairie, MN, USA), micro-IDent^®^plus11 (Hain Lifescience GmbH, Nehren, Germany), and Carpegen^®^ Perio Diagnostik (Carpegen GmbH, Münster, Germany) have also been introduced. They detect several periodontal disease-related pathogens in collected saliva, oral rinse, or plaque based on nucleic acid-based assays. However, many of the microbial assay kits are available in laboratories with some expensive equipment.

### 4.3. Genetic Assay Kits

Analyzing the genetic “susceptibilities” may also help identify or anticipate the potential risk of periodontal disease initiation and progression. It has been known that the polymorphism in the interleukin-1 (IL-1) gene has been shown to be proinflammation-causing periodontal disease [7]. To identify the genetic risk of periodontal disease, multiple test kits have been introduced in the market, including PerioPredict™ (Interleukin Genetics, Inc., Waltham, MA, USA), and MyPerioID^®^ (IL-6 and IL-1; OralDNA Labs, Eden Prairie, MN, USA). However, these tests require significant laboratory equipment or additional time to deliver the sample to the manufacturer’s laboratories for data analysis. Thus, these kits cannot be truly recognized as chairside diagnostics.

Despite several efforts on finding biomarkers, biomarker-based detection has been occasionally applied in dental offices. One reason for the low practical use of biomarker-based techniques is the lack of standardized assays. In addition, there is still no FDA-approved saliva diagnostic test or point-of-care technology for clinical diagnosis of periodontal diseases in the United States (as of September 2020). The good news is that the practical application of biomarkers in periodontal disease diagnosis starts to be discussed in dental research society. In the 2017 World Workshop, the introduction of biomarkers was strongly encouraged as a supportive indicator to identify periodontal disease and to estimate its stage and grade [5]. So far, the specific biomarkers and their thresholds have not been established yet, but we expect they will be incorporated and used in periodontal disease grade assessment as evidence will become available soon. Efforts to accelerate the development of chairside periodontal assay kits or automated biosensors by combining clinically relevant biomarkers with lab-on-a-chip or point-of-care technologies are still active [8,11,158]. Compared to conventional labor-intensive and time-consuming laboratory procedures, the automated chairside periodontal assay methods are believed to provide immediate analysis results related to the disease. Moreover, technologies will evolve toward improving diagnostic sensitivity and accuracy by analyzing multiple analytes simultaneously, although the size of the biosensor is reduced. Furthermore, the ability of chairside analysis of biomarkers for accurate diagnosis and prognosis of the disease will be an important advantage in preventing irreversible damage to periodontal bones and tissues.

## 5. Future Directions

**Figure 3:**
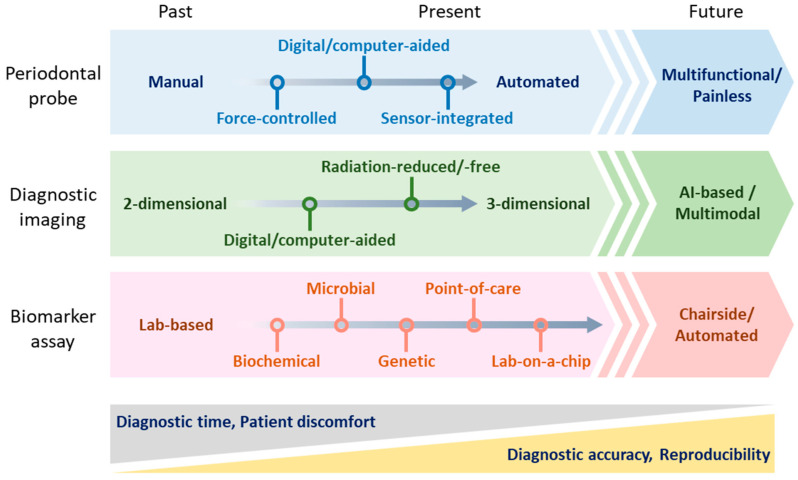
Advancement of chairside periodontal diagnostic tools and their future directions.

Manual periodontal probing and 2D radiography have been the two major diagnostic tools for periodontal disease. Over the years, various new technologies have been incorporated into these two diagnostic tools in attempts to improve their accuracy, reproducibility, speed, and patient comfort (Figure 3). For periodontal probing in clinical examination, early attempts at improvements were derived from the incorporation of advanced mechanical and electrical technologies that enabled accurate and automated assessment. Various sensors (e.g., calculus, temperature, sulfide, and pressure) are being integrated into the periodontal probe platform to provide new information inside the periodontal pocket for a comprehensive analysis. With the development of microfabrication and nanotechnology in the coming years, these sensors are further expected to be miniaturized and integrated into the probe for multifunctional analysis. Furthermore, the approach of non-invasive technology that can quickly and accurately provide 3D information of the diseased area is expected to reduce patient pain and discomfort.

As radiography technology is becoming more developed, the radiograph imaging of the periodontal lesions has gained more significance in periodontal health assessment. In particular, the development of 3D imaging technology like CBCT allowed accurate visualization of bone destruction, which then enabled the precise diagnosis of disease severity and progress. Moreover, since 3D imaging technology is reported to successfully visualize bone and soft tissue using various radio-opaque agents, an accurate 3D map of the periodontal pocket morphology is expected to be possible without painful tactile examination for patients in the future [93]. However, diagnostic radiography exposes a patient to doses of ionizing radiation. As an alternative to radiography, other imaging technologies without ionizing radiation (e.g., ultrasonography or MRI) are being actively studied. It was recently found that a method using MRI can be used for both soft tissue and hard tissue observation, and based on this, it started to gain considerable interest from the periodontal disease research community [137,160]. Lastly, with the integration of state-of-the-art image processing algorithms and artificial intelligence technology, higher accuracy in diagnosis and better prediction in prognosis are expected [98,99,161,162,163].

Although the current standard in the diagnosis of periodontal diseases is still mainly based on clinical examination and diagnostic imaging, recent advances in biomarkers propose a new possibility in early-stage detection and rapid diagnosis. To date, large-scale laboratory assays and many clinical trials have been conducted to identify candidate biomarkers. Moreover, some promising biomarkers have been reported for simultaneous multi-analyte sensing to promote diagnosis accuracy. Based on these developments, it is plausible to believe that automated chairside diagnostic protocols with effective biomarkers will soon be available [158]. Overall, the diagnosis methods for periodontal diseases have continuously advanced with the incorporation of various technologies. It will be no surprise that this trend will continue as we experience more technological advancement and pathological/biological understanding. By embracing new technological developments, clinicians may expand their chairside toolkits to identify, treat, and manage periodontal diseases.

## Figures and Tables

**Table 1:** Comparison between 2D dental radiography and CBCT.

2D Dental Radiography		Techniques	3D CBCT
2D Intraoral X-ray	2D Panoramic X-ray		
~20 lp/mm (Film) [105] 6–15 lp/mm (Digital) [105]	1–4 lp/mm [106]	Resolution (lp/mm)	0.6–2.8 lp/mm [107]
0.005 mSv (Bitewing) [108] 0.035–0.171 mSv (Full-mouth series) [108]	0.003–0.024 mSv [108]	Effective radiation dose	0.011–0.674 mSv (Dentoalveolar CBCT with small and medium field view) [108] 0.030–1.073 mSv (Maxillofacial CBCT with large field of view) [108]
Periapical view- Visualize the root apex, assess severe bone loss Bitewing-Evaluate bone height, assess moderate to severe bone loss	Provides overall view (bird’s-eye view) of the periodontium with the minimized radiation exposure.	Features	Provides axial, coronal, and sagittal multiplanar images with reconstructed form without magnification. Pre-surgical bone quality assessment for osteotomy and implant insertion [109] Assessing crater defects and furcation involvements [110]
2D imaging Superpositions, distortion, and magnification		Imaging method	3D imaging Cross-sectional and volumetric models No image deformation
Lower cost, lower radiation dose, relatively small device		Advantages	High accuracy for detecting periodontal bone defects [111,112]

**Table 2:** Examples of biomarker assay kits in the market.

Biomarker Classification	Sampling From	Product Name	Detecting Target	Detecting Principle	Analyzing in
Biochemical assay	GCF	Periocheck	Neutral proteases	Enzymatic digestion reaction (Colorimetric assays)	Chairside
	GCF	PocketWatch	AST	Enzymatic catalysis reaction (Colorimetric assays)	
	GCF	PerioGard	AST	Enzymatic catalysis reaction (Colorimetric assays)	
	Oral rinse	PerioSafe	aMMP-8	Lateral flow test with digital reader (OraLyzer^®^)	
	GCF	ImplantSafe			
	Oral rinse	SillHa ST-4910	Blood, leukocytes, and protein	Lateral flow test with dual-wavelength reflectometry	
Microbiological assay	Subgingival plaque	Evalusite	*Aa, Pg, Pi*	Sandwich enzyme immunoassay (Colorimetric assays)	Chairside
	Subgingival plaque	BANA-Enzymatic test kit	*Pg, Td, Tf*	BANA hydrolysis reaction (Colorimetric assays)	
	Gums and plaque	OMNIgene ORAL/ OMR-110	Characterization of virus species of all genome type including *Aa, Pg, Pt, Fn, Td, Ec*	DNA hybridization	Company or research laboratory
	Saliva	OMNIgene ORAL/ OM-501, 505			
	Subgingival plaque	Carpegen^®^ Perio Diagnostik	*Aa, Pg, Tf, Td, Fn, Pi*	Real-time qPCR	
	Oral rinse	MyPerioPath^®^	*Aa, Pg, Td, Tf, En, Fn, Pi, Cr, Pm, Ec, Cs*	DNA hybridization	
	Microbiological samples/subgingival plaque	iai Pado Test	*Aa, Pg, Pi, Td, Tf, Fa*	DNA hybridization	
	Subgingival plaque	micro-IDent^®^plus11	*Aa, Pg, Pi, Tf, Td, Pm, Fn, Cr, En, Ec, Cs*	DNA hybridization	
Genetic assay	Cheek swab	PerioPredict™	genes for IL-1	DNA hybridization	Company laboratory
	Oral rinse	MyPerioID^®^ IL-6 or IL-1	genes for IL-6 or IL-1	Genetic polymorphisms detection	

GCF: Gingival crevicular fluid, AST: Aspartate aminotransferase, aMMP: active Matrix metalloproteinase, Aa: Aggregatibacter actinomycetemcomitans, Pg: Porphyromonas gingivalis, Pi: Prevotella intermedia, Td: Treponema denticola, Tf: Tannerella forsythia, Fn: Fusobacterium nucleatum, Ec: Eikenella corrodens, En: Eubacterium nodatum, Fn: Fusobacterium nucleatum/periodonticum, Cr: Campylobacter rectus, Pm: Peptostreptococcus (Micromonas) micros, Cs: Capnocytophaga species (gingivalis, ochracea, sputigena), Fa: Filifactor alocis, IL: Interleukin, qPCR: quantitative polymerase chain reaction.
